# Electrochemical Portable Device for Wastewater Remediation: Evaluating the Efficacy of Zeolites against Ibuprofen Contamination

**DOI:** 10.1021/acsestwater.5c00333

**Published:** 2025-05-14

**Authors:** Antonella Miglione, Dalila Capocotta, Panagiota M. Kalligosfyri, Gabriella Iula, Marco Mancini, Valentina Gioia, Alessandro Frugis, Sossio F. Graziano, Stefano Cinti

**Affiliations:** † Department of Pharmacy, 9307 University of Naples Federico II, 80131 Naples, Italy; ‡ Department of Organic Micropollutants, Acea Infrastructure, 00191 Rome, Italy; § Department of Research, Acea Infrastructure, 00191 Rome, Italy; ∥ Bioelectronics Task Force at University of Naples Federico II, Via Cinthia 21, Naples 80126, Italy; ⊥ Sbarro Institute for Cancer Research and Molecular Medicine, Center for Biotechnology, College of Science and Technology, Temple University, Philadelphia, Pennsylvania 19122, United States

**Keywords:** pharmaceutical contaminants, electrochemical sensing, zeolite-based remediation, wastewater treatment

## Abstract

The increasing prevalence of emerging contaminants, such as pharmaceuticals, pesticides, and industrial chemicals, in wastewater presents significant risks to water quality, ecosystems, and public health. Nonsteroidal anti-inflammatory drugs (NSAIDs), such as ibuprofen, are particularly concerning due to their persistence in wastewater and adverse effects on aquatic environments and biodiversity. Electrochemical sensors have emerged as innovative tools for real-time monitoring of such contaminants, enabling the detection and quantification of trace levels and supporting more effective wastewater management strategies. Among these, zeolites—microporous minerals with high adsorption capacity and ion exchange properties—have demonstrated strong potential for economical, sustainable, and environmentally friendly wastewater remediation, particularly given their ability to be regenerated. In this study, a polyester-based electrochemical sensor for ibuprofen detection was developed, analytically characterized, and validated in wastewater. The sensor achieved a detection limit of 1.6 μg/mL and a repeatability of 8% in wastewater. The remediation system was optimized by evaluating different quantities and exposure times of surfactant-modified and unmodified zeolite-rich tuff powder. Then, the complete setup was successfully tested in the presence of ibuprofen-contaminated wastewater demonstrating a remediation efficiency of 73% using the modified zeolite. The sensor, connected to a portable potentiostat, successfully provided on-site measurements to evaluate the effectiveness of zeolites in wastewater remediation from ibuprofen.

## Graphical abstract

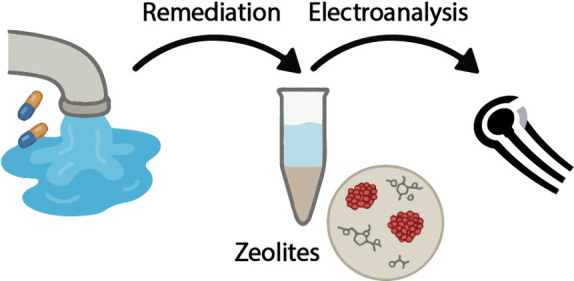

## 1 Introduction

The presence of pharmaceutical contaminants in wastewater has become a significant environmental concern due to their persistence and potential risks to both ecosystems and human health.^1,2^ These contaminants enter water bodies from various sources, including domestic, hospital, and industrial effluents.^3,4^ The challenge posed by pharmaceutical compounds as emerging contaminants lies not only in their diverse chemical structures but also in their ability to persist in the environment. These compounds are often detected in wastewater effluents and surface waters at concentrations that may pose risks to aquatic organisms, even at trace levels.^5,6^ Conventional water treatment methods, originally designed for other pollutants, often prove to be inadequate in effectively removing pharmaceuticals, raising concerns about their long-term impact on water quality and surrounding habitats.^7,8^ Recognizing these risks, human pharmaceuticals were added to the list of emerging contaminants by UNESCO in 2020.^9^ Their detection and elimination have since been incorporated into Goal 6 (Clean Water and Sanitation) of the 2030 Agenda for Sustainable Development (SDG 6).^10^ However, progress toward achieving SDG 6 by 2030 remains significantly off track, highlighting the urgent need for accelerated efforts.^11,12^ To mitigate these challenges, early monitoring of pharmaceutical contaminants together with innovative remediation techniques is essential. Monitoring pharmaceuticals, as emerging contaminants, typically involves analyzing water samples to determine the presence and concentration of specific pharmaceutical compounds. Various analytical methods can be used for this purpose, with the choice of technique depending on factors such as the target pharmaceuticals, required sensitivity, and sample type. Traditional analytical methods primarily include high-performance liquid chromatography (HPLC), liquid chromatography-mass spectrometry (LC-MS), and ultraviolet–visible (UV–vis) spectroscopy.^13–15^ Electrochemical sensing offer a simple, rapid, and selective approach for pharmaceutical detection, with advantages such as low-cost instrumentation and eco-friendly applications.^16,17^ In particular, electrochemical sensors have gained attention for the detection of nonsteroidal anti-inflammatory drugs (NSAIDs),^18,19^ including ibuprofen, naproxen, and diclofenac, due to their high sensitivity, fast response times, and cost-effectiveness.^20–27^ These sensors often incorporate nanomaterials such as carbon nanofibers, graphene quantum dots, and boron-doped ultrananocrystalline diamond (BD-UNCD) to enhance performance, reaching very competitive concentration limits.^26–28^ Despite the advantages—such as low detection limits, minimal sample preparation requirements, and rapid analysis times—these sensors often require complex fabrication processes and may face challenges, particularly interference from complex matrices like wastewater. The concentrations of these kinds of drugs range from 4 to 30 μg/mL in influent wastewater and from 1 to 10 μg/mL in effluent wastewater.^29^ This indicates that while some reduction occurs during treatment, significant amounts still enter the environment; for instance, primary sedimentation techniques have been reported to eliminate only 12–45% of NSAIDs present in wastewater.^30^ Various remediation techniques were developed, each with distinct advantages and limitations. Biological processes, such as biodegradation through activated sludge systems or membrane bioreactors (MBRs), are widely used due to their cost-effectiveness and sustainability.^31^ Advanced oxidation processes (AOPs), including ozonation and photostimulation, offer high degradation efficiency for persistent pharmaceutical contaminants but may lead to the formation of harmful byproducts.^32^ Adsorption using natural materials such as zeolites or carbon-based substances provides an efficient and cost-effective alternative, offering a sustainable approach that can complement traditional remediation methods by improving environmental compatibility and reducing operational costs.^33^ Natural zeolites contained in zeolite-rich tuffs (ZT) can be considered valuable candidates for specific applications from a technological perspective (e.g., adsorption, ion exchange capacity, surface modification, and environmental impacts).^34,35^ These applications take advantage of the unique characteristics of these minerals found in natural deposits, which remain scientifically understudied and underutilized.^36–41^ By integration of electrochemical detection with suitable remediation strategies, it is possible to develop a more comprehensive and efficient approach to monitoring and mitigating pharmaceutical contamination in wastewater. Following this dualistic approach, this study aims to integrate a portable and easy-to-use screen-printed electrode (SPE), capable of selectively detecting ibuprofen as a case study, with ZT-based adsorption systems to evaluate the removal efficiency of ibuprofen from wastewater. By evaluating the performance of ZT (surfactant-modified and unmodified) against ibuprofen contamination using an electrochemical sensing framework, we aim to provide insights into the synergistic benefits of combining these technologies for improved wastewater remediation outcomes. The development of such hybrid systems could provide practical solutions to the challenges associated with pharmaceutical contamination in aquatic environments.

## 2 Experimental Section

### 2.1 Materials

For the fabrication of the screen-printed electrodes (SPEs), conductive inks (Ag/AgCl and graphite) were purchased from Sun Chemicals. Flexible polyester sheets (5HT Autostat) were kindly provided by MacDermid Performance Solutions Italiana, serving as the printing support. Ethanol (denatured, ≥99.5%), acetic acid (glacial, ACS reagent, ≥99.7%), and ammonium acetate (ACS reagent, ≥97%) were purchased by Merck Life Science (St. Louis, MO). Milli-Q water was produced in house to 18 MΩ/cm quality using a Milli-Q (Darmstadt, Germany) EQ 7015 Ultrapure Water Purification System. Ibuprofen sodium salt, unmodified zeolite-rich tuff powder (ZT), and cetylpyridinium chloride (CP-Cl)-modified ZT (ZTm) were collected and prepared in collaboration with the Department of Science and Technology, University of Sannio (Benevento, Italy), and the Department of Earth Sciences, Environment and Resources, Federico II University (Naples, Italy). All of the ibuprofen dilutions were prepared in 0.25 M ammonium acetate buffer (AB) at pH 4.7. The wastewater from drinking water treatment facilities was provided by the Acea Spa (Rome, Italy). All experiments (sensing and remediation) were conducted at room temperature. The pH was fixed at 4.7 for tests performed in acetate buffer, while in wastewater, the pH ranged between 5 and 6, depending on the specific batch, without further adjustment. The sensor’s responses were recorded using the portable Sensit smart potentiostat (PalmSens, Houten, The Netherlands), connected to an Android smartphone. Current responses were recorded and displayed by using the dedicated application PStouch by Palmsens BV.

### 2.2 Zeolite-Rich Tuff Powder Collection

ZT was collected from active quarries operating on the Sorano Formation (Geological Service of the Region of Tuscany, Italy, 2013) and contains more than 50% weight of natural zeolites represented almost exclusively by chabazite.^34^ Tuff powder is produced during quarrying operations and preserves the mineralogical and chemical characteristics of the original rock. As a natural deposit, the amount and type of zeolites depend largely on the geology of the area and the deposit itself, and samples require specific technological characterization to assess the material’s suitability for specific applications.^34,35,40,42–46^

### 2.3 Methods

#### 2.3.1 Preparation of ZT and ZTm Samples

Zeolite-rich tuff powder, in order to be used in interaction with pharmaceutically active molecules, requires a modification that results in a reversal of surface charge.^47^ The ZT sample was then characterized to assess its cation exchange capacity (CEC) and external cation exchange capacity (ECEC) with exchangeable cations (Na^+^, K^+^, Mg^2+^, and Ca^2+^) using the batch exchange method (BEM).^42^ Cation concentrations were estimated in milliequivalents per gram by atomic absorption spectrometry (AAS) on an unmodified sample and a sample treated with a cetylpyridinium chloride (CP-Cl) solution (20 mM).^42^ The surface-modified zeolite-rich tuff powder (ZTm) was then obtained by filtering, washing, and drying the preparation at room temperature. Surface modification was observed by evaluating the ζ potential determined on the nanometric fraction of ZT and ZTm samples using a Zetasizer Ultra apparatus (Malvern).^34^

#### 2.3.2 Fabrication of Screen-Printed Electrodes

The screen-printed electrodes (SPEs) used in this study were prepared in house, as previously reported.^48,49^ The fabrication process involved the use of a semiautomatic screen printer for the printing of all three electrodes (working, reference, and counter electrodes) on flexible polyester sheets, in two steps. First, a silver/silver chloride (Ag/AgCl) ink was used to print the reference electrode and the connections, followed by a thermal treatment at 100 °C for 30 min for solvent removal and ink stability. In the second step, graphite-based ink was applied to print the working and counter electrodes over the Ag/AgCl layer, followed by another thermal treatment step. Polyester was selected as the SPE substrate due to its low cost, mechanical flexibility, chemical stability in wastewater environments, and ease of modification, making it a suitable choice for developing scalable, field-deployable electrochemical sensors.^50,51^ During each measurement step, the SPEs were laminated with an adhesive tape to facilitate liquid handling, ensuring the proper electrical function and sample application on the working electrode.^52^

#### 2.3.3 Electrochemical Detection of Ibuprofen

A stock solution of ibuprofen was prepared by dissolving it in ethanol at a concentration of 1 mg/mL. Serial dilutions were then performed using a 0.25 M acetate buffer (acetic acid/ammonium acetate) at pH 4.7 to obtain final ibuprofen concentrations of 5, 10, 25, 50, 80, and 100 μg/mL. The peak current observed in the DPV measurement is directly proportional to the ibuprofen concentration, enabling its quantitative detection. The sensor’s current response was recorded using differential pulse voltammetry (DPV), as an electrochemical technique, with the following parameters: *E* begin, 0.3 V; *E* end, 1.4 V; *E* step, 0.01 V; *E* pulse, 0.2 V; *t* pulse, 0.02 s; and scan rate, 0.02 V/s. The results were interpreted based on the current response corresponding to each ibuprofen concentration (Figure 1).

**Figure 1:**
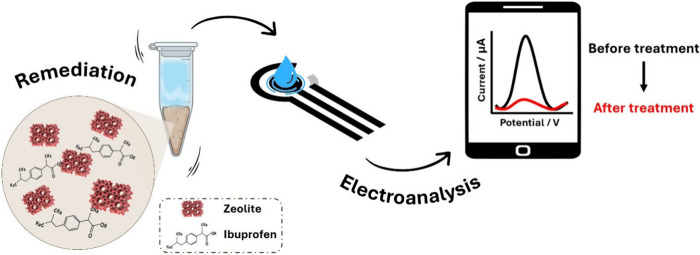
Workflow for electrochemical monitoring of the ZT-based remediation effectiveness against ibuprofen.

#### 2.3.4 Zeolite Remediation Principle and Protocol

For the treatment of the samples, 1 mL ibuprofen-containing solutions (IcS) were prepared. All experiments in this study were conducted using the same batch of ZT and ZTm to ensure consistent material characteristics. Five milligrams of ZT and ZTm were added to IcS, and the mixture was incubated for 60 min under stirring to achieve optimal remediation efficacy. After treatment, the samples were centrifuged, and 100 μL of the supernatant was measured. DPV was used to record the electrochemical responses, and the observed signal decrease after zeolite treatment was correlated with the remediation percentage (*R*%), calculated as [(*I*_control_ – *I*_treated sample_)/*I*_control_] × 100, where *I* is the current intensity response of the electrochemical sensor (Figure 1).

## 3 Results and Discussion

### 3.1 Electrochemical Monitoring of the Zeolite Remediation Principle

The electrochemical detection of ibuprofen at pH 4.7 relies on its oxidation at the electrode surface, typically observed using DPV. At this pH, which is close to ibuprofen’s p*K*_a_ (∼4.9), the analyte exists mainly in its protonated form, influencing its electrochemical behavior.^53,54^ When a potential is applied, ibuprofen undergoes oxidation, resulting in electron transfer to the electrode and the generation of a measurable oxidation peak. The acetate buffer at pH 4.7 provides a stable medium that enhances the solubility and ensures reproducible electrochemical responses.

The remediation of ibuprofen using ZT and ZTm operates primarily through adsorption mechanisms. At pH 4.7, the protonated form of ibuprofen interacts more effectively with the zeolite surfaces. Zeolites utilize a combination of ion exchange and hydrophobic interactions to adsorb ibuprofen, where the protonated molecules can replace other cations within the zeolite’s structure. The modification with CP-Cl further enhances this process by introducing cationic surfactant groups that improve electrostatic attraction and increase hydrophobic capacity, thereby increasing the affinity for ibuprofen molecules.^34^

### 3.2 Optimization Studies of Ibuprofen Electrochemical Detection

For optimal detection of ibuprofen using the proposed flexible screen-printed electrochemical sensor, an investigation of the analyte’s working solution was necessary. Due to ibuprofen’s poor solubility in aqueous solutions,^54^ the stock solution was prepared at a concentration of 1 mg/mL in ethanol. First, the effect of three different working solutions at different pH values was evaluated: phosphate-buffered saline (PBS) at pH 7.24, acetate buffer (AB) at pH 6.5, and acetate buffer (AB) at pH 4.7. To determine the optimal working solution, a known ibuprofen concentration of 100 μg/mL was tested. As expected, the acetate buffer at pH 4.7 provided the highest compound stability over time and the most reproducible measurements, as shown in Figure 2.

**Figure 2:**
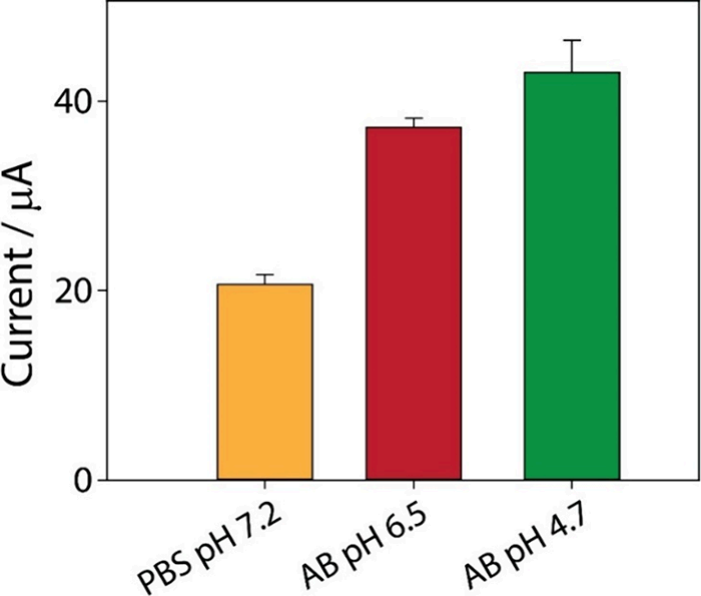
Optimization study of the working solution in the presence of 100 μg/mL ibuprofen. All experiments were performed in triplicate. DPV parameters: *E* begin, 0.3 V; *E* end, 1.4 V; *E* step, 0.01 V; *E* pulse, 0.2 V; *t* pulse, 0.02 s; and scan rate, 0.02 V/s.

The choice of a pH 4.7 buffer was further supported by its ability to maintain an optimal environment for drug stability while maximizing the sensitivity of the analytical technique. At this pH, the carboxyl functional group of ibuprofen remains in its protonated form, facilitating electrochemical oxidation through the electron transfer. Consequently, this solution was selected as the optimal condition for further optimization of the analytical system.^54^

### 3.3 Analytical Evaluation of the Developed Sensor in a Buffer Solution

The analytical performance of the optimized method was evaluated using increasing concentrations of ibuprofen (IBP), initially dissolved in ethanol and subsequently diluted in acetate buffer at pH 4.7, within the range of 5–100 μg/mL. Each calibration point was measured in triplicate to ensure statistical robustness and assess signal consistency. Blank samples (i.e., a buffer solution without ibuprofen) were analyzed under the same conditions to determine baseline signal levels. The resulting calibration curve, shown in Figure 3, is described by the equation *y* = 0.39*x* – 2.01 (*r* ^2^ = 0.98). The *y*-axis represents the measured current response (microamperes), while the *x*-axis corresponds to the ibuprofen (IBP) concentration in micrograms per milliliter. The limit of detection (LOD) was calculated as 3 times the standard deviation (σ) of the blank divided by the slope (*S*) of the calibration curve (LOD = 3σ/*S*), yielding a value of 1.4 ± 0.2 μg/mL. The limit of quantification (LOQ) was calculated as 10 times the standard deviation (σ) of the blank divided by the slope (*S*) of the calibration curve (LOQ = 10σ/*S*) and resulted in a value of 4.8 ± 0.2 μg/mL. The repeatability of the developed sensor was also assessed in terms of the relative standard deviation (RSD%; *n* = 6), which was calculated as the ratio between the standard deviation and the mean value of the measurements at a concentration of 25 μg/mL, multiplied by 100. This parameter provides an evaluation of the system’s repeatability, which was determined to be 6.3%.

**Figure 3:**
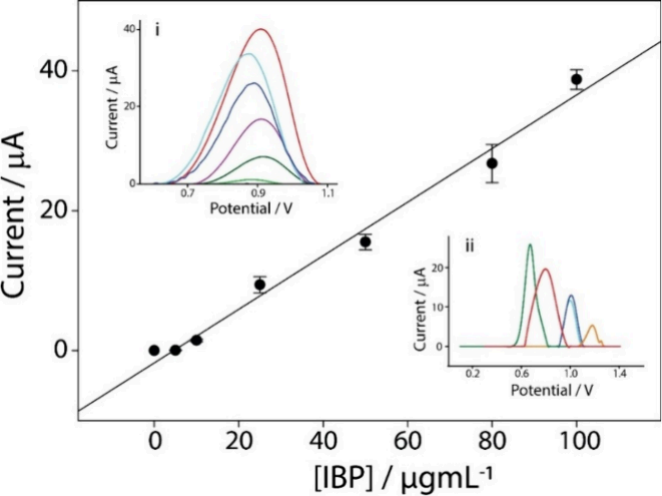
Calibration curve obtained in AB at pH 4.7, at increasing IBP concentrations from 0 to 100 μg/mL. The insets show (i) DPV curves in the same concentration range and (ii) a selectivity study. The selectivity study was performed in the presence of 10 μg/mL IBP (red line) compared with 10 μg/mL solutions of paracetamol (blue), uric acid (dark blue), ascorbic acid (orange), and carbamazepine (green), all dissolved in AB at pH 4.7. All of the experiments were performed in triplicate. DPV parameters: *E* begin, 0.3 V; *E* end, 1.4 V; *E* step, 0.01 V; *E* pulse, 0.2 V; *t* pulse, 0.02 s; and scan rate, 0.02 V/s.

To investigate the selectivity of the sensor, four potential interferents commonly found in the reference matrix, i.e., wastewater, were evaluated.^55,56^ The selected interferents included paracetamol, carbamazepine, uric acid, and ascorbic acid, each tested at a concentration of 10 μg/mL under the same experimental conditions. As shown in inset ii of Figure 3, the selected interferents do not affect the behavior of ibuprofen, in the same potential range, ensuring the selectivity of the sensors.

### 3.4 Analytical Evaluation of the Developed Sensor in Spiked Wastewater

The analytically characterized and optimized sensor was also evaluated in a wastewater sample to assess the effect of the wastewater matrix effect. The analytical performance of the optimized method was assessed using increasing concentrations of ibuprofen (IBP), initially dissolved in ethanol, and then diluted in the wastewater sample. To avoid diluting the tested matrix, the linearity of the system was directly tested in the wastewater sample without further dilution. The wastewater used, depending on the aliquot, had a pH ranging between 5 and 6, where ibuprofen exists as a protonated form and is thus available for oxidation. The ibuprofen concentration ranged from 0 to 100 μg/mL. The resulting calibration curve in wastewater gave the equation *y* = 0.27*x* – 1.41, with an *r* ^2^ value of 0.97 (Figure 4). Subsequently, the LOD and LOQ were calculated as described previously, resulting in values of 1.6 ± 0.2 and 5.2 ± 0.2 μg/mL, respectively. Finally, the repeatability of the sensor in the wastewater application, as expressed by the RSD%, showed a value of 8.4% (*n* = 6). As expected, the calibration slope in wastewater was lower than that in buffer, indicating some signal attenuation likely due to matrix complexity. However, the correlation remained strong, and the LOD in wastewater was only slightly higher (1.6 μg/mL vs 1.4 μg/mL in buffer). The inclusion of blanks also confirmed that no significant electrochemical interference occurred in either the buffer or wastewater matrices. To further assess the analytical performance in real samples, recovery experiments were conducted by spiking wastewater with known ibuprofen concentrations (10, 25, and 50 μg/mL). Recovery rates ranged between 91% and 99%, demonstrating reliable detection, even in the presence of matrix constituents.

**Figure 4:**
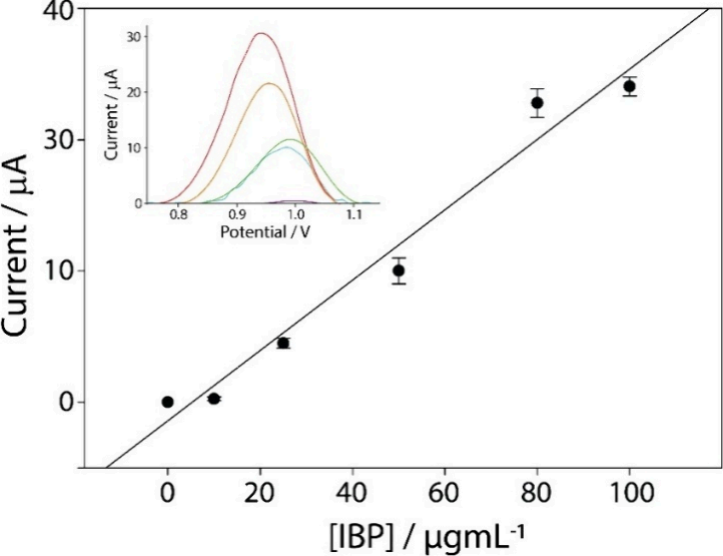
Calibration curve obtained in the presence of an increasing target concentration from 0 to 100 μg/mL prepared in the wastewater sample. The inset shows the DPV curves. All of the experiments were performed in triplicate. DPV parameters: *E* begin, 0.3 V; *E* end, 1.4 V; *E* step, 0.01 V; *E* pulse, 0.2 V; *t* pulse, 0.02 s; and scan rate, 0.02 V/s.

### 3.5 Optimization Studies for Ibuprofen Remediation

To optimize the amount of ZT samples required for ibuprofen remediation, three different quantities of ZTm were tested (1, 2, and 5 mg) and incubated with 1 mL of a 25 μg/mL IBP solution. Each sample was stirred for 60 min to enhance the interaction between the sample and the target compound. The results showed that the remediation percentages (*R*%) for the different ZTm amounts were as follows: 71% for 1 mg, 72% for 2 mg, and 91% for 5 mg (Figure 5A). Subsequently, 5 mg, given the highest percentage of ibuprofen removal, was chosen to continue the optimization of the incubation time.

**Figure 5:**
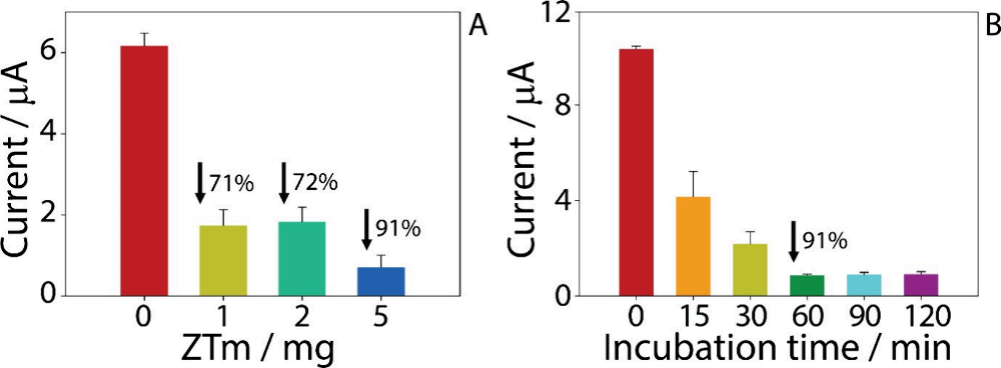
(A) Optimization of ZTm quantity in the presence of 25 μg/mL ibuprofen, incubated for 60 min. A 0 mg ZTm portion represents the untreated control sample of 25 μg/mL ibuprofen. (B) Effect of incubation time on the remediation process, using 5 mg of ZTm and a 25 μg/mL IBP solution, incubated for 0, 15, 30, 60, 90, and 120 min. The remediation percentages (*R*%) are reported in both figures. All experiments were performed in triplicate.

The incubation time was evaluated from 0 to 120 min, using a solution containing 25 μg/mL ibuprofen. The aim of this study was to evaluate the impact of incubation time on the adsorption efficiency, determining how the contact duration between the modified zeolite and the analyte influences the remediation capacity. As shown in Figure 5B, the incubation time significantly affects the remediation process. A substantial reduction in ibuprofen concentration was already observed within the first 15 min (*R*% = 60%), with maximum removal achieved at 60 min (*R*% = 91%). Based on these results, 60 min was selected as the optimal incubation time to test the system in real matrices, as discussed in the following section.

### 3.6 Remediation Efficiency Evaluation

The efficiency of the remediation by using zeolite-rich powders was investigated by using both modified and unmodified samples. The aim of this study was to evaluate the adsorption capacity of zeolite-rich natural powdered samples in the working solution, i.e., acetate buffer at pH 4.7, and in the wastewater samples, using the developed sensor system. A 25 μg/mL IBP solution was incubated with both unmodified (ZT) and surfactant-modified (ZTm) samples. Specifically, three 1 mL solutions were prepared: the first served as the control solution, containing 25 μg/mL ibuprofen; the second and third contained 25 μg/mL ibuprofen incubated with 5 mg of unmodified (ZT) and modified zeolite-rich samples (ZTm), respectively. These solutions, prepared in either buffer or wastewater, were mixed for 1 h before electrochemical analysis of the ibuprofen concentration. In both cases, the remediation efficacy showed a significant difference in the responses, suggesting a notable impact from the interaction with the modified zeolites. The resulting remediation percentage was found to be 44% for treatment with the unmodified sample (ZT) and 94% for treatment with the surfactant-modified one (ZTm) (Figure 6A). Treatment with zeolite-rich powder led to a reduction in the detected signal intensity, with a greater reduction observed for the surfactant-modified zeolites compared with the unmodified ones. In the presence of uric acid, paracetamol, ascorbic acid, and carbamazepine, the ibuprofen remediation efficiency slightly decreased from 94% to 89%, suggesting moderate competitive adsorption. While the sensor maintained reliable selectivity, only carbamazepine showed partial overlap in the detection range but was not effectively removed by the modified zeolite. This behavior can be due to its neutral, hydrophobic nature and limited interaction with the modified zeolite surface, which is more effective toward polar or anionic species such as ibuprofen.^57,58^ Similarly, an *R*% of 19% was noted in wastewater samples, while a higher *R*% of 73% was achieved with ZTm, demonstrating the efficiency of the remediation material in real matrix application (Figure 6B).

**Figure 6:**
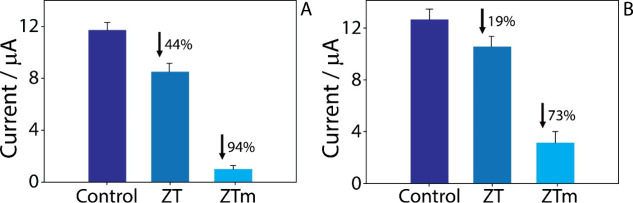
Remediation efficiency tested in (A) acetate buffer pH 4.7 and (B) wastewater in the presence of 25 μg/mL IBP, incubated for 60 min with 5 mg of zeolites (ZT) and surfactant-modified zeolites (ZTm). The remediation percentages (*R*%) are reported in both panels in comparison with the control (dark blue histograms). All experiments were performed in triplicate.

The surfactant-modified zeolite (ZTm) facilitated ibuprofen removal through a combination of electrostatic attraction between the drug’s anionic form and the positively charged pyridinium head groups, hydrophobic interactions with the CP-Cl alkyl chains, and π–π stacking between aromatic rings, contributing to the observed 73% remediation efficiency.^34,59^ The large surface area and high microporosity of these natural samples contribute significantly to their effectiveness in removing ibuprofen from aqueous solutions under these conditions, making them efficient tools for environmental remediation efforts targeting pharmaceutical pollutants such as ibuprofen at slightly acidic pH levels typical of wastewater treatment systems or natural waters. The results showed that treatment with zeolite-rich powders causes a significant reduction in the signal intensity associated with the oxidation of ibuprofen, with a greater reduction observed for treatment with ZTm, even in the real matrix, where the remediation process may be less effective due to the presence of other interferent species that can be adsorbed by the zeolites. The achieved remediation efficiency aligns well with values reported for other ibuprofen removal techniques, such as adsorption using activated carbon that typically shows removal efficiencies ranging from 60% to 90%, depending on surface area, pore structure, and pretreatment methods.^60,61^ Similarly, advanced oxidation processes (AOPs), such as ozonation or UV/H_2_O_2_, can achieve higher removal (>90%) but often require larger energy inputs or complex setups or generate transformation byproducts.^62^ Bioremediation strategies involving specific bacterial strains show variable results, with efficiencies between 40% and 80%, often requiring longer treatment times and specific conditions.^63^ Compared with these methods, our approach offers a cost-effective, low-energy, and reusable alternative using a naturally available zeolite, modified with a common surfactant. The in situ monitoring capability provided by the integrated electrochemical sensor further enhances the system’s practical applicability. The simplicity and affordability of both the polyester-based electrochemical sensor and the modified natural zeolite support the potential for scalable and cost-effective deployment. Given their respective reusability and adaptability, this integrated system may be suitable for decentralized environmental monitoring and remediation strategies, particularly in low-resource settings or in small-scale wastewater treatment facilities.

### 3.7 Green Metrics of the Developed Method

In the field of analytical chemistry, green chemistry is a fundamental consideration when planning laboratory procedures.^64^ To comprehensively assess the environmental sustainability and practicality of our developed method, we evaluated it using three recognized green assessment tools: AGREEprep, the Click Analytical Chemistry Index (CACI), and the Modified Green Analytical Procedure Index (MoGAPI). First, the AGREEprep metric was applied to assess the greenness of the sample preparation step. This tool evaluates 10 essential criteria, such as waste generation, energy consumption, integration with the analytical procedure, sustainability, and operator safety.^65^ Our method achieved a score of 0.79, which reflects a strong green profile. Given that AGREEprep scores range from 0 (poorest performance) to 1 (optimal performance or no sample preparation required), this result highlights the low environmental impact, reduced resource usage, and minimal sample handling required by our procedure, characteristics that align well with green analytical chemistry principles. To complement this evaluation, we assessed the practicality and feasibility of our method using the CACI tool.^66^ This user-friendly software provides a composite score based on method sensitivity, simplicity, availability of equipment, and overall practicality. Our use of homemade electrochemical sensors, which are inexpensive but also commercially accessible, real-time measurement, and the lack of a requirement for sample pretreatment helped reduce both the economic and environmental footprint of the method. The compact design, ease of use, and capability for handling multiplexed water-based matrices, particularly in the context of emerging contaminants, contributed to the method achieving a CACI score of 81. A score of more than 75% is classified as highly practical, confirming the field applicability and user-friendliness of our system. Finally, we evaluated the overall environmental performance of the method using MoGAPI.^67^ This recently developed metric allows for a comprehensive assessment of analytical method greenness, incorporating features such as online real-time sampling, the lack of pretreatment, minimal solvent use, solvent reuse, and the application of sustainable sensors. Our method attained a MoGAPI score of 85, which classifies it as an “excellent green” method (≥75). This high score underscores the method’s minimal environmental impact, operational efficiency, and alignment with modern green analytical approaches. Taken together, the outcomes of these three evaluation tools (AGREEprep (0.79) (Figure 7A), CACI (81) (Figure 7B), and MoGAPI (85) (Figure 7C)) are largely consistent and support the overall greenness, practicality, and applicability of our analytical method. These complementary assessments not only demonstrate the sustainable nature of the procedure but also validate its potential for real-world environmental monitoring and remediation applications.

**Figure 7:**
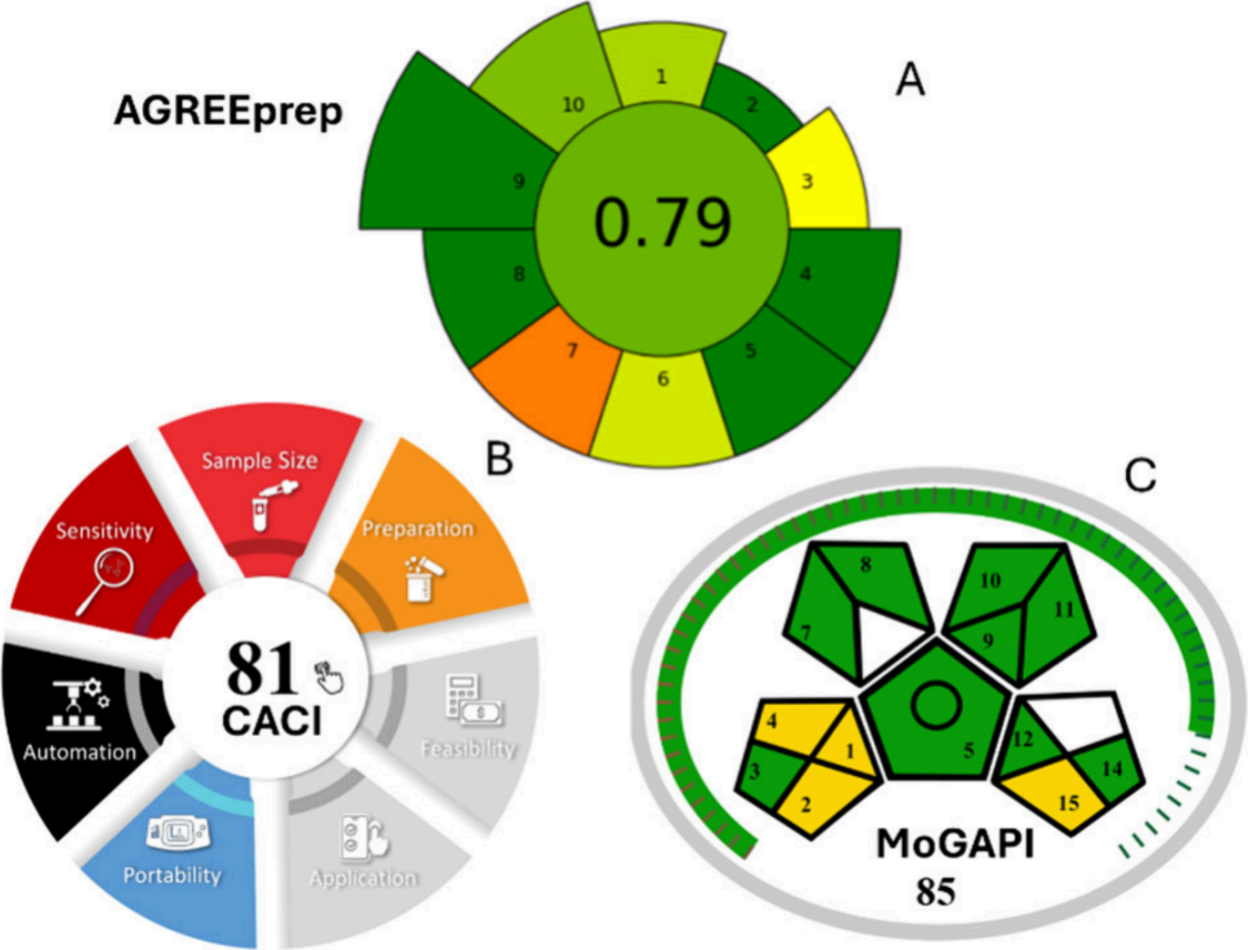
Visual representation of the green metric assessment of the developed method using (A) the AGREEprep scale, (B) the CACI metric, and (C) the MoGAPI tool. All three tools illustrate the method’s favorable performance, highlighting its strong environmental sustainability and practical applicability.

## 4 Conclusions

The contamination of water sources by pharmaceuticals such as ibuprofen poses a growing environmental and public health challenge. To tackle this issue, surfactant-modified zeolite-rich samples (ZTm) were employed as an advanced remediation material, significantly enhancing the adsorption efficiency compared to that of unmodified samples. Additionally, a portable, screen-printed electrochemical sensor was developed to enable real-time monitoring of the remediation process, offering a rapid and on-site detection method that eliminates the need for complex laboratory procedures. Its high sensitivity and portability make it a valuable tool for wastewater treatment facilities, ensuring regulatory compliance before discharge. The sensor demonstrated excellent selectivity for ibuprofen, minimizing interference from other wastewater constituents, and achieved detection limits in the parts per million range, allowing precise assessment of remediation efficiency. The surfactant modification further improved interactions of the zeolites with organic pollutants, increasing the adsorption capacity and overall removal performance. Under the optimized conditions, the modified zeolites achieved a removal efficiency of 91% in a buffer solution and 73% in real wastewater, significantly outperforming unmodified zeolites. These results highlight the effectiveness of surfactant-modified zeolites in treating complex wastewater matrices. Moreover, the literature suggests that surfactant-modified zeolites can maintain adsorption performance over multiple uses with minimal surfactant release under controlled conditions.^34,57,58,68–70^ Future work will focus on assessing the regeneration efficiency and long-term environmental impact of the CP-Cl-modified zeolite system. Beyond industrial applications, this approach holds promise for broader environmental monitoring. The green metric evaluations confirm that the proposed method is not only environmentally sustainable but also highly practical. Its strong performance across AGREEprep, CACI, and MoGAPI highlights its potential for use in real-world environmental monitoring applications. By integrating highly efficient remediation with real-time electrochemical detection, this study presents a scalable and practical solution for addressing pharmaceutical contamination in water systems, contributing to sustainable environmental management and public health protection.
